# Liquid-State and Solid-State Properties of Nanotube/Polypropylene Nanocomposites Elaborated *via* a Simple Procedure

**DOI:** 10.3390/nano3010173

**Published:** 2013-03-06

**Authors:** Arrate Huegun, Mercedes Fernández, Juanjo Peña, María Eugenia Muñoz, Antxon Santamaría

**Affiliations:** 1 Polymer Science and Technology Department and Polymer Institute POLYMAT, Faculty of Chemistry and J. M. Korta Building, University of the Basque Country (UPV/EHU), P.O. Box 1072, E-20080 San Sebastian, Basque Country, Spain; E-Mails: arrate.huegun@ehu.es (A.H.); mercedes.fernandez@ehu.es (M.F.); mariaeugenia.munoz@ehu.es (M.E.M.); 2 Department of Physics of Materials, Faculty of Chemistry, University of the Basque Country (UPV/EHU), P.O. Box 1072, E-20080 San Sebastian, Basque Country, Spain; E-Mail: juanjo.pena@ehu.es

**Keywords:** carbon nanotubes, rheology, crystallization, conducting polymers, rheological percolation, electrical percolation

## Abstract

Non-modified Multiwalled Carbon Nanotubes (MWCNT) and polypropylene (PP) in absence of compatibilizer have been chosen to elaborate MWCNT/PP nanocomposites using a simple melt-mixing dispersing method. Calorimetry results indicate little effect of MWCNTs on crystallinity of PP, revealing not much interaction between nanotubes and PP chains, which is compatible with the employed manufacturing procedure. In any case, a hindering of polymer chains motion by MWCNTs is observed in the molten state, using oscillatory flow experiments, and a rheological percolation threshold is determined. The percolation limit is not noticed by Pressure-Volume-Temperature (PVT) measurements in the melt, because this technique rather detects local motions. Keeping the nanocomposites in the molten state provokes an electrical conductivity increase of several orders of magnitude, but on ulterior crystallization, the conductivity decreases, probably due to a reduction of the ionic conductivity. For a concentration of 2% MWCNTs, in the limit of percolation, the conductivity decreases considerably more, because percolation network constituted in the molten state is unstable and is destroyed during crystallization.

## 1. Introduction

In their seminal review of 2006 [1], Moniruzzaman and Winey stated that the fabrication methods of carbon nanotubes (CNT)/polymer nanocomposites were focused on improving CNT dispersion, because a better dispersion in the polymer matrixes was found to improve properties. Under this premise, more generally accepted six years ago than today, many efforts have been devoted to improving the dispersion of carbon nanotubes, striving to avoid the natural trend of CNTs to form bundles, owed to the combination of van der Waals forces and high polarizability. The procedures used to prevent bundled arrangements, involve, on one hand, the development of performing mixing methods, such as dispersion-reaction (including polymerization at CNT surface), melt mixing and others, described by Grady [2], and on the other hand, reducing the surface energy of CNT, either by chemical covalent functionalization or physically, by wrapping of surfactants, solvent free modification by tertiary phosphines and other methods, exhaustively described by Bose *et al.* [3]. More recently, a combination of anionic and cationic surfactants at different molar ratios has been reported to be an adequate method to obtain performing surface-modified CNTs [4]. The use of compatibilizers has also been investigated, as a route to improve CNT dispersion in polymer matrixes [5,6,7,8,9,10,11,12,13]. Particular attention has been paid to the use of maleic anhydride grafted polypropylene as a compatibilizer for CNT/isotactic polypropylene (PP) nanocomposites [5,11,12,13,14].

Therefore, the research carried out to improve CNT dispersions has been remarkable. However, one of the most interesting properties associated to the use of carbon nanotubes, *i.e.*, the electrical conductivity, has not been observed to enhance with a perfect dispersion or total absence of CNT bundles. Rather to the contrary, thermal treatments of the nanocomposites in the molten state, in quiescent state or under small shear, have shown that the agglomeration processes enhance dramatically the electrical conductivity, through the formation of a conductive network of interconnected clusters [15,16,17,18,19,20,21,22,23,24,25]. In particular, MWCNTs dispersion and agglomeration, under thermal treatment, in amorphous matrixes, such as polystyrene (PS) and polycarbonate (PC), and semicrystalline matrixes, e.g., PP, have been investigated. The correlation of the dispersion/agglomeration state with the electrical conductivity is analyzed in depth in reference [15]. It is seen that the final value of the electrical conductivity is independent of the initial dispersion state, provided that the same thermal treatment is applied. Needless to say, these results open a quite facile route to elaborate electrically conductive nanocomposites, which has been explored to prepare MWCNT/PP nanocomposites [15,24,26]. Certainly, we have to recognize that the efforts to reach a good CNT dispersion, modifying MWCNTs and/or using compatibilizers, to fabricate MWCNT/PP nanocomposites do not necessarily bring better electrical conductivity results [27,28,29,30,31] than when neat components are adequately melt mixed and submitted to annealing in the molten state. In this regard, the results obtained by Lee *et* *al.* [12] are significant. In a paper published in 2007, the authors combine MWCNT modification and compatilibilizers, obtaining a high electrical conductivity (above 10^−3^ S/cm) in MWCNT/PP nanocomposites, with MWCNTs chemically functionalized and maleic anhydride grafted styrene-ethylene/buthylene-styrene (MA-g-SEBS). However, as an example of how good electrical results are not only obtained using complex procedures, in 2008 the same research group reported what, to our knowledge, is the highest electrical conductivity ever published for MWCNT/PP nanocomposites: 10^−1^ S/cm [5], with 3% MWCNT and 5% MWCNT concentrations prepared without compatibilizer and non-modified carbon nanotubes. The role played in these results by the thermal treatment implied in the compression molding should not be discarded.

In this paper, the rheological behavior in the molten state, and the thermal and electrical properties in the molten and in the solid state of a MWCNT/PP nanocomposites prepared in a very simple melt mixing way, are investigated. The obtained original results are organized to respond to the following issues, scarcely treated in the literature: (a) Effect of MWCNTs on the crystallization process of PP, when neither compatibilizers, nor functionalized carbon nanotubes are used; (b) Rheological percolation observed in the terminal or flow region of dynamic viscoelastic measurements; (c) Capacity/inability of PVT measurements to detect a percolation threshold; (d) Effect of the MWCNT concentration on the electrical conductivity increase observed during annealing in the molten state; (e) Effect of the MWCNT concentration on the electrical conductivity decrease observed during crystallization.

## 2. Results and Discussion

### 2.1. Thermal Properties

**Figure 1:**
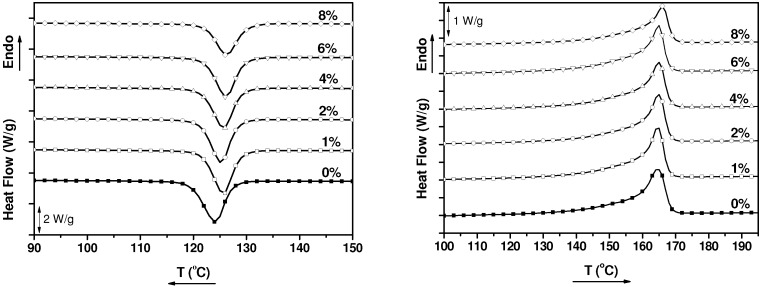
Differential Scanning Calorimetry (DSC) cooling and heating scans of neat PP and MWCNT/PP nanocomposites obtained at 10 °C/min.

Differential Scanning Calorimetry (DSC) heating and cooling curves of neat PP matrix and MWCNT/PP nanocomposites are displayed in Figure 1.

A slight increase of the crystallization temperature is observed with the addition of carbon nanotubes (Table 1). The measurements were repeated three times giving an error of ±0.5 °C corroborating that the crystallization temperature, *T*_c_, augments up to 2.3 °C.

**Figure 2:**
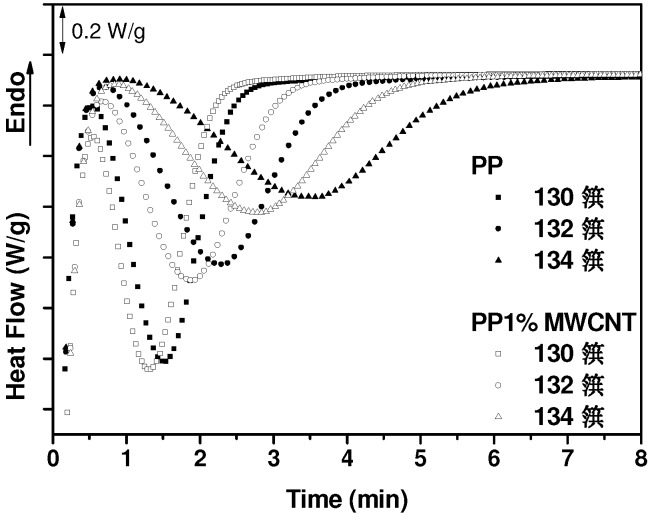
Isothermal DSC results obtained with pure PP and 1 wt.% MWCNT/PP nanocomposite at the indicated temperatures.

To confirm the effect of MWCNTs on the crystallization process, isothermal DSC measurements were carried out. The results obtained with the nanocomposite which contains 1 wt.% MWCNT are presented in Figure 2. A nucleating effect of the MWCNTs is noticed by a reduction of the crystallization time, this reduction becoming more evident as the temperature of the isothermal measurement is increased.

**Table 1:** Thermal data of neat polypropylene (PP) and Multiwalled Carbon Nanotubes (MWCNT)/PP nanocomposites obtained from DSC results of Figure 1.

Sample	*T*_c_ (°C)	*T*_m_ (°C)	*X*_c_ (%)
**PP**	124.2	164.5	38
**PP1%** **MWCNT**	125.6	164.6	39
**PP2%** **MWCNT**	125.2	164.9	40
**PP4%** **MWCNT**	125.8	164.9	38
**PP6%** **MWCNT**	126.2	164.9	40
**PP8%** **MWCNT**	126.5	165.9	34

This is compatible with a lower viscosity, which favors the mobility of the polymer chains towards the crystallization surface and thus accelerates the crystallization process [32]. On the other hand, the evaluated crystallization degree of pure PP and the nanocomposites, also included in Table 1, reveals that the crystallization degree varies between 38% and 40% aleatory for all the samples, except for 8 wt.% MWCNT nanocomposite, which shows a lower value of 34%. This decrease of the crystallization degree observed at the highest MWCNT concentration is probably a consequence of the steric hindrance, which reduces the chain ability to crystallize, induced by the considerable amount of carbon nanotubes [4].

Many papers have been published on changes in the crystallization kinetics and nucleating effect of carbon nanotubes in semi-crystalline polymers in general [33] and PPs in particular [4,28,30,34,35,36]. The recent reviews of Bikiaris [36] and Grady [33] offer an overview of the effect of nanotubes in PP crystallization. The nucleation mechanism of polypropylene on the surface of SWCNTs and MWCNTs has been explained assuming that PP macromolecules are adsorbed and partly wrapped around the CNT, allowing to form nuclei with crystallographic c-axis perpendicular to the axis of CNTs [37]. Actually, dispersions reached at the nanoscale will favor wrapping of PP, which is compatible with the observed higher crystallization increase with the better dispersion, reported in the literature [14,38,39]. Actually, the degree of interaction between polymer and CNT plays the most important role, since weak CNT-polymer interaction reduces polymer-wrapping around CNTs. This has been pointed out by Zeng *et al.* [40] for the case of MWCNT/Polyoxymethylene nanocomposites, demonstrating the effect of the surface energy of CNT and the wettability of polymer on the crystallization degree. In the case of MWCNT/PP nanocomposites, the very low polarity of pure PP is an impediment for any interaction with carbon nanotubes. To avoid this and to favor dispersion of MWCNTs in PP matrixes using melt-mixing procedure, a double strategy has been developed in recent years. MWCNTs have been functionalized attaching oxygen containing groups, such as carboxyl, carbonyl or hydroxyl groups [6,7,12] and, on the other hand, coupling agents consisting of graft or block copolymers (*i.e.*, maleic anhydride grafted polypropylene) have been used as a third component [12,14,41].

Within this context, the little effect of MWCNTs on the crystallization process of PP, as is noticed in Figure 1, Figure 2, Table 1, probably reflects the small degree of interaction between the nanofiller and the polymer in our nanocomposites. This is a logical consequence of using commercial components, such as non-modified MWCNTs and PP, which are based on the leitmotiv of this work. In any case, our polypropylene presumably contains a nucleating agent, and it could be expected that the effect of MWCNTs on the crystallization process would be more evident in a PP free of this kind of agents [33,42].

### 2.2. Analysis of the Molten State: Oscillatory Flow and PVT Measurements

The study of the nanocomposites in the molten state is of crucial importance, because, as has been remarked specially by the group of Alig and Pötschke [16,17,18,19,25,26,43,44,45,46], a rearrangement of carbon nanotubes/polymer chains network can take place above *T*_m_ (or at approximately *T*_g_ + 100 °C in amorphous polymers), leading to the so called *secondary agglomeration* [15]. Besides its scientific interest, this thermally driven process is very relevant from a practical point of view, because brings about a striking improvement of the electrical conductivity of the nanocomposite.

**Figure 3:**
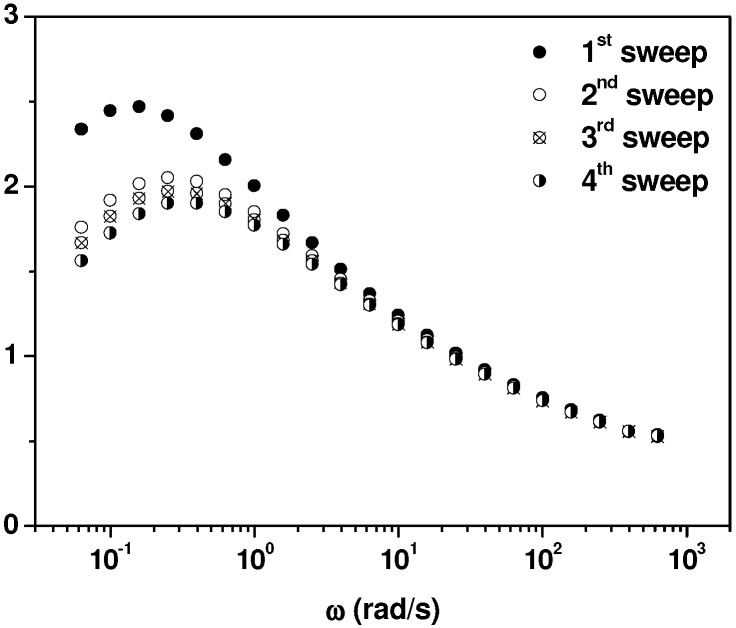
Consecutive frequency sweeps: loss tangent as a function of frequency for 2 wt.% MWCNT/PP nanocomposite at 180 °C at different times (1^st^ sweep at 30 min, 2^nd^ sweep at 100 min, 3^rd^ sweep at 130 min, 4^th^ sweep at 200 min).

We first investigate the effect of time on the dynamic viscoelasticity at *T* = 180 °C, to detect any change in the MWCNTs-PP chains interactions. The evolution of the elastic modulus (*G’*) with time in the molten state of MWCNTs nanocomposites with PP and polycarbonate (PC) respective matrixes, has been reported in the literature [17,20,21], revealing an enhancement of *G’* for microscale dispersions [17] or a reduction of *G’* for well-dispersed nanocomposites [20,21]. The increase of the elastic modulus is reported to be a consequence of the formation of a combined MWCNTs-polymer network. Our microscopy (see Figure 10) and thermal results indicate an “initial aggregation” state or microscale dispersion, so dynamic viscoelastic results can help to analyze the eventual evolution of this primary structure. The most significant result is presented in Figure 3, which shows the effect of time on the loss tangent (tan δ) plots as a function of frequency (ω). 

An example of the effect of nanoparticles/polymer chain interactions on tan δ *versus* frequency plots is shown in the literature for phenoxy/nanoclay nanocomposites [47]: Instead of a continuous increase of tan δ as frequency tends to zero (due to the mobility of the whole chain in the flow viscoelastic zone), a tan δ maximum (tan δ)_Max_ appears at low frequencies. This maximum marks a limit in the mobility of the polymer chains, owing to the topological effect of the nanoparticles. Our results in Figure 3 constitute an example of the presence of a (tan δ)_Max_ relaxation for MWCNT/PP nanocomposites, in contrasts with neat polymers that present a continuous increase of tan δ. A progressive diminution of the height of (tan δ)_Max_ and a shift to lower frequencies is observed, as time increases with different sequential sweeps. The shift of the maximum signifies that the reduction of the mobility is noticed at higher frequencies (equivalent to shorter times) as time increases, which denotes more effective topological interactions between MWCNTs and PP chains.

After reaching stable values of the dynamic viscoelastic functions at *T* = 180 °C, the variation of the elastic (*G’*) and loss modulus (*G’’*) with frequency for the different compositions is analyzed, to evaluate the rheological percolation threshold of our thermally treated nanocomposites.

**Figure 4:**
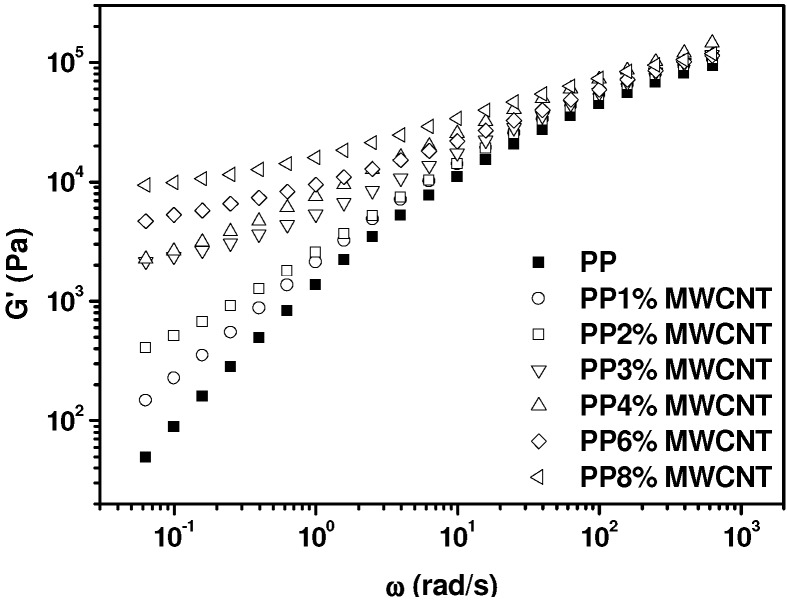
Storage modulus against frequency at 180 °C for neat PP and MWCNT/PP nanocomposites.

**Figure 5:**
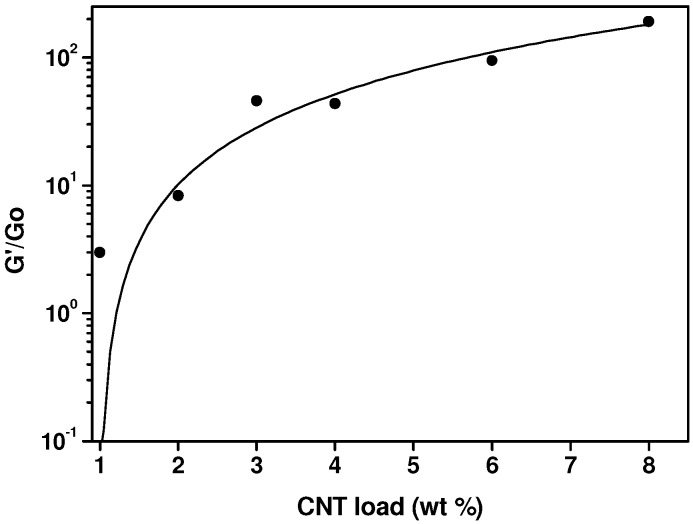
Normalized storage modulus at *T* = 180 °C as a function of MWCNT concentration fitted to the equation of the statistical percolation theory (see text).

The results shown in Figure 4 indicate that as the concentration of MWCNTs increases, in particular above 2 wt.% MWCNTs, the elastic modulus tends to level off. This is considered a symptom of the interactions between nanoparticles and polymer chains that bring about a percolated network, which suppresses flow. The statistical percolation theory [48] predicts the dependence of a particular property (*X*) on filler concentration (Φ as a scaling law: *X* = *X*_0_ (Φ − Φ*_c_*)*^t^*, where Φ*_c_* is the percolation threshold and *t* is an adjustable parameter. This statistical percolation theory is applied considering the values of *G’* taken at the lowest values of the frequency (0.0628 rad/s) divided by the lowest value of the elastic modulus for neat PP, *G’*(0.0628 rad/s)/*G*_0_ value. The experimental data fitted to the scaling law of the percolation theory are presented in Figure 5. The obtained percolation threshold is Φ*_c_* = 1, and the exponent *t* = 1.5.

PVT measurements offer complementary information to dynamic viscoelastic tests in the analysis of polymer chain dynamics. For instance, the lattice-hole model developed by Simha and Somcynsky [49] introduces the concept of a temperature-dependent hole fraction, *h*, related with free volume, which can be obtained from PVT measurements and has been correlated with transport properties [50,51]. Certainly, the eventual effect of nanoparticles on hole fraction or on free volume is a subject of interests, but very few paper refer to PVT measurements of nanocomposites. The efforts of Utracki [52,53,54,55,56,57,58] have been mostly directed to adapt and apply the PVT equations of state to nanocomposites obtained from dispersions of organoclays, in amorphous and semicrystalline polymer matrixes. The existence of a percolation threshold for the constitution of a nanoparticle/polymer chain network is not treated in these works, probably because PVT measurements are not able to detect this physical fact. Significantly enough, in Reference [55] Utracki remarks that dynamic viscoelastic measurements reveal the formation of structures of interacting nanoclay platelets in a 5% nanoclay/Polyamide 6 nanocomposite, but not for 2% concentration. However, their PVT results do not show any qualitative change between 2% and 5% nanoclay compositions.

**Figure 6:**
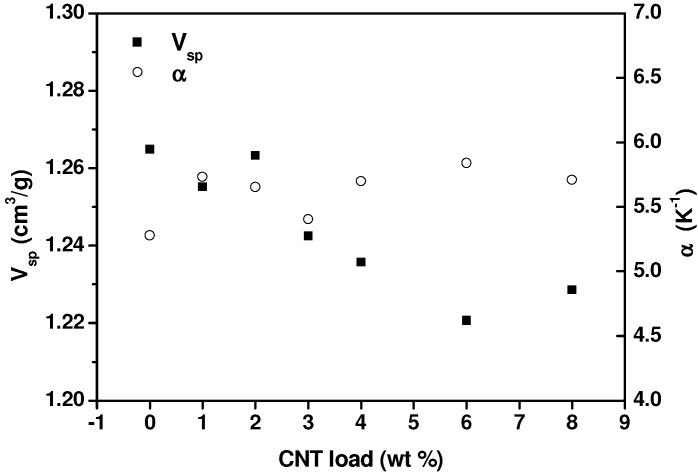
Specific volume (filled symbols) and thermal expansion coefficient (empty symbols) results at *T* = 180 °C and *P* = 200 bar for PP and MWCNT/PP nanocomposites of indicated MWCNT concentration.

The difficulty of applying equations of state to hybrid materials impedes us to determine the hole-fraction and free volume of our nanocomposites. However, the obtained thermodynamic coefficients, such as the thermal expansion coefficient and the compressibility coefficient (not shown), do not reveal the existence of any percolation threshold. In Figure 6, the eventual effect of the nanotube concentration on specific volume and thermal expansion coefficient is analyzed. To the difference of oscillatory flow results presented in Figure 4, which show a qualitative change in *G’* curves (from convex to concave) when passing from 2 wt.% to 3 wt.% MWCNT concentration, PVT data do not follow any particular trend or alteration. This reflects the inability of this technique to detect percolation, because no particular difference is noticed in PVT results below and above the rheological percolation threshold determined by oscillatory flow experiments (Figure 5).

From these results it can be concluded that the very local polymer chain motions involved in the creation of free volume as temperature increases, are not affected, as least in our case, by the presence of MWCNTs. This is actually an expected result if one considers the effect of frequency on the dynamic viscoelastic functions, in Figure 3, Figure 4. At high frequencies (*i.e.*, above 10^2^ rad/s), the effect of MWCNTs is dramatically reduced and the percolation threshold cannot be detected, because the hindering effect of MWCNTs only affects to the motion of the whole chain (observed only in the terminal zone, at low frequencies).

### 2.3. Electrical Conductivity Measurements in the Molten State, during Crystallization and in the Solid State

**Figure 7:**
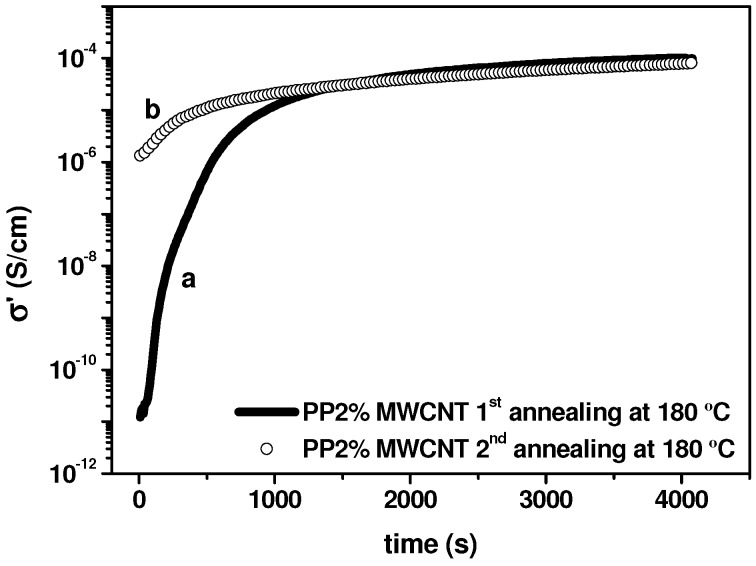
Electrical DC conductivity as a function of time during a quiescent annealing at 180 °C for 2 wt.% MWCNT/PP nanocomposite: (**a**) 1^st^ annealing; (**b**) 2^nd^ annealing.

Following the *modus operandi* established by Alig and Pötschke (see for instance Reference [15]), we first investigate the effect of time on electrical conductivity, at *T* > *T*_m_, to correlate this result with the constitution of the secondary agglomeration, mentioned in Section 2.2. As an example, the electrical conductivity variation with time in the quiescent state at *T* = 180 °C for 2% MWCNT/PP nanocomposite is presented in Figure 7. This temperature is sufficiently high to facilitate the motion of the polymer chains in the terminal viscoelastic zone. The observed conductivity increase is very similar to the behavior depicted by Alig *et al.* [25,26], confirming the rearrangement of MWCNTs in the matrix to form conductive aggregates. The mechanism for electrical conduction, implies that within the agglomerates the distances between the nanotubes are sufficiently short to allow electron tunneling, and also implies the formation of a conductive network of interconnected agglomerates [21].

We remark that in the first annealing, which is initiated taking the molded sample (see Experimental Section), the conductivity augments seven orders of magnitude, from 10^−11^ S/cm to 10^−4^ S/cm. However, in the second annealing, which is carried out after the annealed sample has been cooled from 180 °C to 50 °C at a rate of 1 °C/min, the reached final conductivity is the same, but the initial conductivity is five orders of magnitudes (10^−6^ S/cm face to 10^−11^ S/cm) higher than in the first annealing. Considering the results presented in Figure 21 of reference [15], our results of Figure 7 indicate that the starting point of the second annealing is a more agglomerated dispersion than that we have at *t* = 0 in the first annealing. This can be explained assuming that the secondary aggregation state, reached at the end of the first annealing, is partially preserved on cooling, in particular during crystallization. The effect of annealing time on the electrical conductivity is particularly dramatic for 2% MWCNT/PP nanocomposite, probably because this composition is relatively close to the rheological percolation threshold and in the limit of the electrical percolation threshold in the molten state, as it is seen below. Actually, for a concentration of 4 wt.% MWCNT the effect of annealing is less severe, since the observed increase of the electrical conductivity is only o approximately two orders of magnitude.

**Figure 8:**
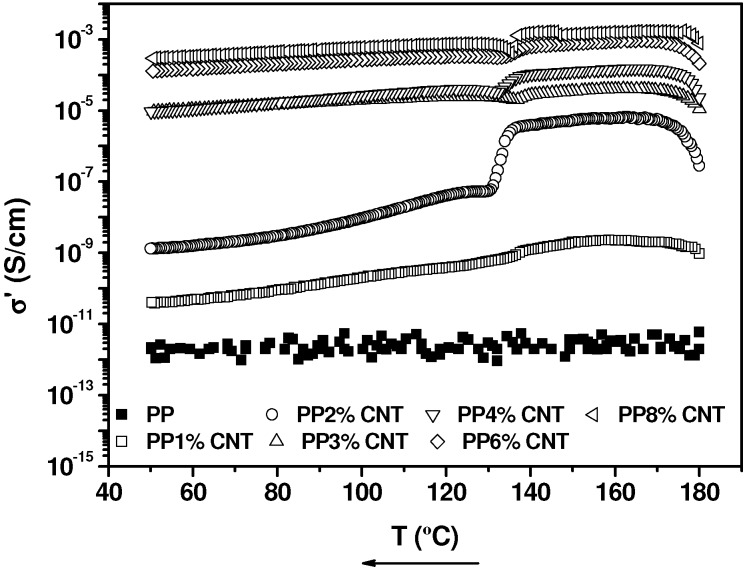
Electrical conductivity (DC) of MWCNT/PP nanocomposites during the cooling sweep from 180 °C up to 50 °C. The effect of temperature on the ionic conductivity of PP could not be adequately analyzed because of limitations of our equipment.

The analysis of the electrical conductivity as a function of frequency for different MWCNTs concentrations in the molten state, at *T* = 160 °C (not presented), shows a practically frequency independent DC conductivity (except for pure PP and 1% MWCNT/PP nanocomposite) and a power law frequency dependency, σ(*f*) = *A υ^S^*, which stands for AC conductivity, above a certain critical frequency value. The evolution of the conductivity of the nanocomposites taken at a frequency of 20 Hz, which corresponds to the constant conductivity zone for the samples that have DC conductivity, on cooling from the melt, is displayed in Figure 8.

We first remark that during crystallization, at approximately 125 °C–130 °C, an abrupt conductivity decrease is observed, similarly to that reported in the literature [59]. In a recent paper about the increase of electrical conductivity of MWCNT/Polyurethane (PUR) nanocomposites during crystallization, we summarize literature results of different nanocomposites (Table 1 of reference [59]). Two hypotheses are contemplated for the case of a conductivity decrease during crystallization: (a) Reduction of the ionic conductivity of the nanocomposite due to a reduction of the amorphous phase of the matrix, considered by Alig *et al.* [26] and (b) Destruction of the conductive pathways, because conducting nanoparticles are expelled from the crystalline phase, contemplated by Pang *et al.* [60]. The observation of Figure 8 leads to remark that the conductivity decrease is much more severe in the case of 2% MWCNT concentration than in the rest of the sample: A reduction of two orders of magnitude face to less than one. To investigate this result, which is linked to the proximity of 2% MWCNT concentration to the rheological percolation limit (see above), in the continuation we analyze the electrical percolation threshold in the molten state, at *T* = 160 °C, and in the solid state, at *T* = 50 °C. The obtained results are helpful to disclose the origin of the observed conductivity changes during crystallization.

**Figure 9:**
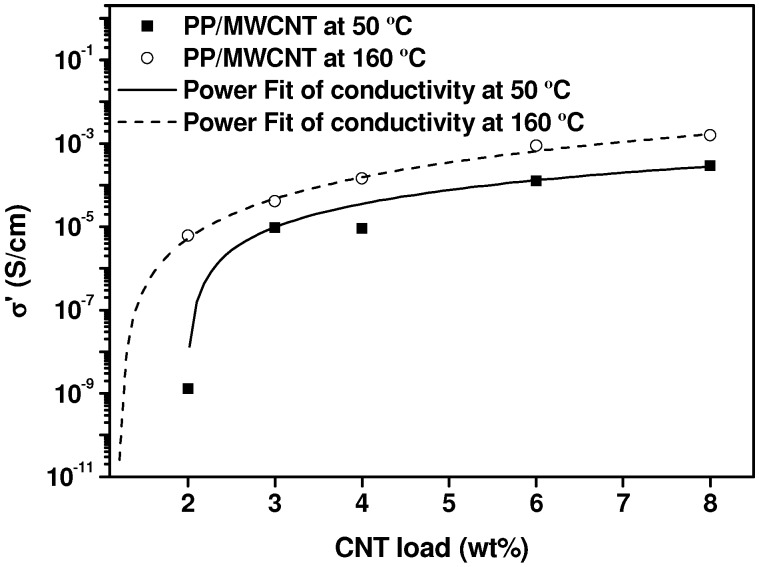
Electrical conductivity (DC) as a function of MWCNT content at 50 °C and 160 °C fitted to the equation of the statistical percolation theory (see text).

The effect of MWCNTs concentration on the electrical conductivity of the nanocomposites at respective temperatures of 160 °C and 50 °C is presented in Figure 9. The corresponding fittings to the equation of the theory of percolation, *X* = *X*_0_ (Φ − Φ*_c_*)*^t^*, (see section 2.2), taking X as the electrical conductivity (σ’), give the following values for the adjusting parameters: Φ*_c_* = 1.2 and *t* = 2.7 for *T* = 160 °C and Φ*_c_* = 2 and *t* = 1.8 for *T* = 50 °C. Therefore, the percolation threshold is significantly higher after nanocomposites have crystallized.

In particular, the concentration of 2% MWCNTs is above the percolation threshold estimated in the molten state (Φ*_c_* = 1.2%), but is just in the percolation threshold (Φ*_c_* = 2%) estimated in the solid state. In view of these results, we can consider that the percolating network, associated to the secondary agglomeration reached in the molten state for 2% MWCNT/PP nanocomposite, is rather unstable and is destroyed during crystallization process, because of crystals growth. However, for concentrations clearly above the percolation threshold in the molten state, Φ*_c_* = 2%, such as 3% up to 8% MWCNT shown in Figure 8, the constituted network is sufficiently strong not to be destroyed during crystallization and the conductivity decrease is much more smaller, approximately one order of magnitude. This reduction may be due to a reduction of the ionic conductivity of the nanocomposite, compatible with a reduction of the amorphous phase of the polymer matrix, as proposed by Alig *et al.* [60]. These results are different, but not contradictory, with those obtained with MWCNT/PUR nanocomposites [60], which show a conductivity decrease during crystallization for Φ < Φ*_c_*, only compatible with a ionic conductivity reduction, but a conductivity increase for Φ > Φ*_c_*, that is explained by a reorganization of the conductivity network inside the aggregates, brought about by crystal growth.

Our electrical conductivity results come to confirm that maintaining the nanocomposites for a certain time at temperatures at which the polymer chain mobility is allowed (*T* > *T*_m_ for semicrystalline polymers) brings about high electrical conductivities, with respect to the observed values in polymer nanocomposites, although a reduction of approximately one order of magnitude should be expected during solidification process. From a practical point of view, this constitutes a reliable strategy to obtain semiconductor polymers, face to complex elaboration procedures, based on modifying the natural lack of interaction between carbon nanotubes and polymer chains, which do not offer necessarily better results.

## 3. Experimental Section

### 3.1. Materials

Considering the scope and the aims of the paper, a significant characteristic of the filler and the polymer matrix used in this work is that both are non-modified, which indicates that the simplest commercially available MWCNTs and polypropylene are employed in the elaboration of the nanocomposites.

The investigated isotactic polypropylene (Moplen EP340K; *M*_w_ = 440,000 g/mol, *M*_w_/*M*_n_ = 4.4) was purchased from Basell Polyolefines Company (Ferrara, Italy), whereas Multiwall Carbon Nanotubes (MWCNT), which have specified diameters of *D* = 30–50 nm, lengths between 10 and 20 μm and purity greater than 95%, were supplied by Cheap Tubes Inc. (Brattleboro, VT, USA).

### 3.2. Preparation of PP/MWCNT Nanocomposites

A very single procedure was used to disperse the MWCNTs in the iPP matrix. Before the melt-mixing process, polymer powder was prepared from pellets using a Mill Retsch® ZM 200 (Retsch Technology, Düsseldorf, Germany) and both, polymer powder and MWCNTs, were properly dried (PP 80 °C at vacuum, 2 h; MWNT 100 °C, 2 h). The polymer and MWCNTs were stirred to obtain a homogeneous mixture. The powder mixture was blended in a Haake Mini-Lab twin-screw extruder (Thermo Electron Corp., Hamburg, Germany). The mixing was processed at *T* = 180 °C and 100 rpm using a counter-rotating screw configuration for 10 min. Extrudate was cooled at room temperature and then pelletized.

### 3.3. Characterization

#### 3.3.1. Transmission Electron Microscopy (TEM)

**Figure 10:**
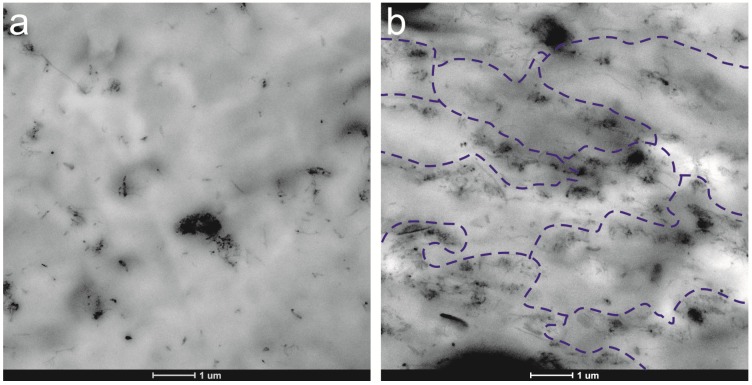
TEM micrographs of nanocomposites: (**a**) 2 wt.% MWCNT/PP; (**b**) 4 wt.% MWCNT/PP. Tentative pathways formed by nanotube agglomerates are marked by dashed blue lines in the case of 4 wt.% MWCNT/PP.

The MWCNT/PP nanocomposites were trimmed using an ultramicrotome device at −60 °C (Leica EMFC6; Leica Microsystems, Vienna, Austria) equipped with a diamond knife. The ultrathin sections (100 nm) were placed on 300 mesh copper grids. The surfaces were observed by TEM (TECNAI G2 20 TWIN; FEI Company, Eindhoven, the Netherlands), operating at an accelerating voltage of 200 KeV in a bright-field image mode. As could be expected considering our experimental procedure, the interaction forces between MWCNTs keep them forming aggregates that can be seen in TEM microphotographs shown in Figure 10.

Assuming that two length scales of dispersion should be considered in carbon nanotube/polymer nanocomposites [61], the nanoscale and the microscale, our case corresponds to the latter. The aggregates, which respond to what Alig *et al.* [15] define as “initial aggregates”, are homogeneously dispersed. TEM results display a two dimensional cut of a 3D structure and, therefore, it is not possible to see through going pathways formed by agglomerates, directly in Figure 10. The obtained image notwithstanding, allows drawing a tentative percolation pathway for nanotube agglomerates.

#### 3.3.2. Differential Scanning Calorimetry (DSC)

The PP and MWCNT/PP nanocomposite samples with different content of carbon nanotubes were subjected to DSC analysis under, both, non-isothermal and isothermal conditions using a Q2000 differential scanning calorimeter (TA Instruments, New Castle, DE, USA) with nitrogen as the purge gas to avoid any oxidation. The temperature scale of the calorimeter was calibrated with indium. All samples were weighed (about 7 mg) and enclosed in an aluminum pans and empty aluminum pan was also used as a reference.

In order to determine the peak crystallization temperature (*T*_c_), each sample was fast heated to 210 °C at a rate of 10 °C/min, held for 5 min to erase any thermal history and then cooled down at a rate of 10 °C/min to. After that, the sample was held for 5 min at −70 °C and heated up to 200 °C at a constant rate of 10 °C/min to analyze the peak melting temperature (*T*_m_) as well as the enthalpy of melting (∆*H*_m_). The specific heat flow from the melting peak was corrected for the mass of polypropylene in the nanocomposite. The PP matrix crystallinity in the nanocomposites was calculated with using the melting enthalpy value for 100% crystalline polypropylene (∆*H*_m100_ = 209 J/g; α-form [62]).

For an isothermal analysis samples were heated to 210 °C and kept at this temperature for five minutes. After this period, samples were rapidly cooled at the crystallization temperature (130 °C, 132 °C, 134 °C) and maintained at the corresponding temperature for the time necessary for complete crystallization.

#### 3.3.3. Rheological Characterization

The dynamic viscoelastic behavior of compression molded samples (*T* = 180 °C for 5 min) was investigated using an ARG2 rheometer (TA instruments) with parallel-plate geometry (25 mm). Dynamic frequency sweep experiments were conducted in molten state at 180 °C from 628 rad/s to 0.0628 rad/s within viscoelastic linear regime.

#### 3.3.4. Pressure-Volume-Temperature (PVT) Measurements

Pressure-Volume-Temperature behavior was analyzed in a PVT (PVT 100 Haake) apparatus of the piston die technique made by Haake (PVT 100 Haake). Measurements were carried out in an isobaric cooling mode procedure at 200 bar using a cooling rate of 5 °C/min.

#### 3.3.5. Conductivity Measurements

The ARES Rheometer (TA Instruments) Dielectric Analysis option (DETA) with an Agilent E4980A Bridge (Agilent, CA, USA) was used to determine the electrical conductivity. Electrodes were 25 mm diameter stainless steel plates and the measurements were performed on compression molded samples of 0.4 mm thickness.

Two types of test were carried out using a voltage of 1 V and a frequency of 20 Hz: Time sweep at 180 °C and temperature sweep from 180 °C to 50 °C at a cooling rate of 1 °C/min. Before the temperature sweep, specimens were held at 180 °C along 5 min.

Dielectric data were recorded for each sample as a function of time or temperature depending on the test. The real part of the conductivity as a function of the angular frequency (*ν*), is calculated from the imaginary part of the dielectric constant ε׳׳(*ν*) through the relation:

[σ'(υ)=2πε_0_ε׳׳(υ)] (1)

where ε_0_ = 8.85 × 10^−12^ F/m is the vacuum permittivity.

The variation of the conductivity with frequency is described by:

[σ(*f*)=σ_DC_ +Aυ^S^] (2)

where *ν* is the frequency, A is A constant depending on temperature and *S* is an adjustable parameter.

This behavior has been considered as the Universal Dynamic Response, as it is applicable to AC electronic and ionic conduction in disordered solids and does not depend on the details of the disorder. DC value is calculated from the frequency independent conductivity data associated to the low frequency regime.

## 4. Conclusions

MWCNT/PP nanocomposites have been elaborated with non-modified carbon nanotubes and in absence of compatibilizers using a single melt-mixing dispersing method. The MWCNTs form microscale aggregates that can be seen in TEM microphotographs, and DSC results show that the crystallization temperature, as well as crystallization degree of PP, is little affected by MWCNTs. This is probably a consequence of the facile elaboration procedure chosen *ad hoc* for the aims of the research.

The interaction of the polymer chains with the MWCNTs is noticed in the molten state, using small amplitude flow experiments. The hindering effect of carbon nanotubes on PP chains motion is observed in the terminal viscoelastic zone, at low frequencies, as a tan δ relaxation, which stands for the suppression of the viscoelastic flow response, is detected. This is linked to the marked trend of the elastic modulus to level off, as MWCNTs concentration increases. The application of the equation of the statistical theory of percolation leads to a rheological percolation threshold of Φ*_c_* = 1. PVT results obtained in the molten state for different MWCNTs concentrations do not reflect any particular change above and below Φ*_c_* = 1, which is explained considering that the hindering effect of MWCNTs, associated to rheological percolation, only affects to the motion of the whole chain whereas PVT measurements only detect very local polymer chain motions.

Keeping the nanocomposites in the quiescent state at 180 °C gives rise to a very significant electrical conductivity increase, which is particularly huge in the case of 2% MWCNT/PP nanocomposite, since the conductivity passes from 10^−11^ S/cm at *t* = 0 min to 10^−4^ S/cm after one hour. During crystallization from the melt, the conductivity decreases approximately in one order of magnitude for MWCNTs concentrations which are above the electrical percolation threshold evaluated in the molten state, Φ*_c_* = 1.2%. We assume that this is due to a reduction of the ionic conductivity of the nanocomposite, compatible with a reduction of the amorphous phase of the polymer matrix. However, for 2% MWCNT/PP, which corresponds to the limit of the percolation threshold, the reduction of the electrical conductivity during crystallization is much more severe than for the other compositions: more than two orders of magnitude, face to one order. This result is explained considering that the percolation network constituted in the molten state is relatively unstable and is destroyed during crystallization process, because of crystals growth.

Our electrical conductivity results show that maintaining the nanocomposites for a certain time at temperatures at which the polymer chain mobility is allowed (*T* > *T*_m_ for semicrystalline polymers), brings about high electrical conductivities. From a practical point of view, this constitutes a reliable strategy to obtain semiconductor polymers using a simple procedure.
